# Safety and surgical outcomes of sodium fluorescein-guided pediatric brain tumor surgery: a retrospective before-and-after single-center cohort study

**DOI:** 10.3389/fsurg.2026.1875948

**Published:** 2026-09-29

**Authors:** Christian Ott, Alexander Hebeler, Tommaso Araceli, Barbara Greiner, Katharina Rosengarth, Martin Proescholdt, Nils-Ole Schmidt, Sebastian Siller

**Affiliations:** 1 Department of Neurosurgery, University Medical Center Regensburg, Regensburg, Germany; 2 Universitat Regensburg, Regensburg, Germany; 3 Department of Radiology, University Medical Center Regensburg, Regensburg, Germany

**Keywords:** fluorescein-guided surgery, pediatric neuro-oncology, sodium fluorescein, tumor resection, YELLOW 560 filter

## Abstract

**Purpose:**

Sodium fluorescein (SF) used with a YELLOW 560-nm filter may improve intraoperative visualization of contrast-enhancing brain tumors, but pediatric evidence remains limited. We assessed the feasibility, safety, and surgical outcomes of SF-guided resection in a single-center pediatric cohort.

**Methods:**

We retrospectively analyzed pediatric brain tumor resections performed between 2015 and 2023 using a before-and-after design based on the institutional introduction of SF. SF-guided procedures were compared with historical white-light controls. Inclusion required a contrast-enhancing intracranial tumor considered amenable to gross total resection (GTR). SF was administered intravenously at 2 mg/kg. The primary endpoint was radiographically confirmed GTR within 72 h; operative duration, postoperative course, complications, and follow-up outcomes were secondary exploratory endpoints. Exploratory multivariable logistic regression for GTR included SF use, age, sex, and supra- vs. infratentorial location. Operative duration was compared using a two-sided Mann–Whitney *U*-test.

**Results:**

Among 60 procedures in 50 patients, 32 were performed with SF and 28 without SF. GTR was achieved in 93.8% of SF-guided and 71.4% of non-SF procedures (absolute difference, 22.4 percentage points; Fisher's exact *p* = 0.035). In exploratory multivariable analysis, SF use was associated with GTR (adjusted odds ratio 5.890, 95% confidence interval 1.091–31.080; *p* = 0.039). In an unadjusted exploratory analysis, operative duration was lower with SF [median 191.5 min (IQR 148.25–264.25) vs. 249.5 min (IQR 184.00–306.75); *U* = 303, *p* = 0.032]. No SF-related adverse reactions occurred. Fluorescence was documented in 20/32 SF-guided procedures and was positive in 17/20 of those cases.

**Conclusion:**

Low-dose SF was safe and feasible and was associated with higher GTR rates and lower operative duration. However, the historical-control design, limited number of non-GTR outcomes, heterogeneous tumors, baseline differences, unequal follow-up, and residual confounding preclude causal or long-term oncological conclusions. Prospective validation is required.

## Introduction

Pediatric brain tumors are the most common solid malignancies in childhood and a leading cause of cancer-related mortality in patients younger than 20 years, accounting for approximately 20%–25% of all pediatric cancers and include highly heterogeneous entities (1, 2). Despite major advances in multimodal therapy, maximal safe surgical resection remains a cornerstone of treatment for many pediatric brain tumors. However, in the molecular era, the prognostic relevance of the extent of resection is increasingly recognized as entity- and subgroup-dependent. While complete resection may be curative in selected circumscribed low-grade tumors such as pilocytic astrocytoma, the survival benefit of further resection is less uniform in other entities, including medulloblastoma, where molecular subgroup affiliation substantially modifies the prognostic impact of residual tumor (3–5). Whenever anatomically and functionally feasible, achieving gross total resection (GTR) can reduce recurrence risk and, for low-grade tumors such as pilocytic astrocytomas, may be curative while sparing children from potential neurocognitive or endocrinological sequelae of adjuvant therapies (5).

However, pediatric brain tumor surgery is often performed in complex regions such as the posterior fossa, fourth ventricle, brainstem vicinity, and deep midline structures. Narrow corridors, developing and delicate neurovascular anatomy, and vulnerability to perioperative physiological fluctuations increase operative complexity (6). In addition, pediatric brains are more prone to deformation, which can reduce the reliability of navigation and intraoperative imaging and complicate the identification of true tumor boundaries (7).

In this context, fluorescence-guided surgery has emerged as an adjunct to improve intraoperative visualization. Sodium fluorescein (SF) is a hydrophilic dye that accumulates in areas of blood–brain barrier disruption, mirroring gadolinium enhancement on MRI and providing real-time fluorescent contrast between enhancing tumor tissue and surrounding brain parenchyma (8–10). Unlike 5-ALA, which depends on tumor-specific metabolism, SF primarily relies on disruption of the blood–brain barrier; many common enhancing pediatric tumors are therefore candidates for SF-based visualization. SF can be administered at low doses (typically 1–5 mg/kg) shortly before or after dural opening; at these doses, adverse reactions are rare, although anaphylactoid reactions have been reported (11, 12).

In adult neurosurgery, SF-guided resection is established for high-grade gliomas and cerebral metastases, among other contrast-enhancing lesions (13–15). Pediatric data remain limited and are largely based on small cohorts; de Laurentis and colleagues and Göker and Kırış reported feasibility and fluorescence patterns under the YELLOW 560 filter, and our group previously reported SF use in pediatric posterior fossa lesions (16–18). Because direct comparison data are scarce, the impact of SF on resection rates and operative outcomes in pediatric patients remains unclear. We therefore systematically analyzed the comparison between SF-guided and white-light resections in our institute, focusing on feasibility and safety, extent of resection, and operative duration, and short-term postoperative and follow-up outcomes.

## Methods

### Patients

The study was approved by the local institutional review board (IRB) of the University of Regensburg (Z-2015-0478-9) and was conducted in accordance with the principles expressed in the Declaration of Helsinki. Informed consent was obtained from all legal representatives (parents) of the patients for both the off-label use of SF and study participation, in accordance with the standards of the local IRB.

This retrospective study used a before-and-after design based on the institutional introduction of sodium fluorescein in pediatric brain tumor surgery during the study period. Accordingly, SF-guided resections were compared with historical white-light controls from the pre-SF era rather than with a contemporaneous control group. Inclusion criteria were patients under the age of 18 years with a gadolinium-enhancing supra- or infratentorial intra-axial cerebral neoplasm for which gross total resection was considered surgically feasible and intended based on preoperative imaging and interdisciplinary surgical assessment. Tumors in which complete resection was not considered safely achievable because of eloquent-area involvement or other anatomical constraints were not included.

### Pre- and postoperative clinical and neuroradiological assessment

All patients (and cases) had previously undergone a gadolinium-enhanced MRI scan. Each patient received contrast-enhanced MRI within 72 h after surgery to rule out hemorrhage and to confirm the extent of resection. A blinded neuroradiologist distinguished two categories: no residual tumor tissue = GTR and residual tumor tissue = subtotal resection (STR).

Baseline characteristics were collected, and neurological assessment was performed in all patients preoperatively at admission for planned surgical treatment, at discharge after surgery, and at every follow-up time point. Patients follow-up was initially performed every 3 months and continued until the time of death. The detailed neurological examination included motor function assessment, evaluation of sensory function and pain level, and gait and vegetative assessments according to the age of the patient.

### Surgical protocol

All patients in the sodium-fluorescein group were administered a weight-adjusted dose of 2 mg/kg body weight of SF. The reduced dosage compared with that used in our adult patients (5 mg/kg body weight) was implemented as a precautionary measure for this potentially vulnerable patient population. If *en bloc* surgery, the preferred method of tumor resection, was not feasible, tumors were removed in a piecemeal fashion under fluorescence visualization with a YELLOW 560 filter integrated into the microscope. Whenever feasible, all areas showing sodium fluorescein enhancement were resected.

Additional intraoperative adjuncts, including neuronavigation, intraoperative ultrasound, and neuromonitoring, were used selectively according to the individual surgical indication and were not applied as part of a standardized protocol. The use of adjunctive technologies was compared between the SF and non-SF groups using Fisher's exact test.

Fluorescence intensity was retrospectively extracted from the operative reports and categorized as intense, medium, slight, or absent, according to the terminology used by the operating surgeon. If the operative report did not contain an assessment of fluorescence intensity, the variable was classified as missing and was not interpreted as absent fluorescence.

Because fluorescence intensity was incompletely documented and only two SF-guided procedures resulted in subtotal resection, no inferential per-case analysis of fluorescence grade vs. extent of resection was performed.

Six primary surgeons performed procedures during the study period. Surgeon-specific case numbers were small and temporally clustered. Therefore, no formal surgeon-level comparison was performed, and the surgeon was not included as an additional covariate in the multivariable model.

### Statistical analysis

Statistical analyses were performed using SigmaPlot for Windows version 11 and IBM SPSS Statistics. Continuous variables were summarized as means and standard deviations and, when distributions were skewed, as medians and interquartile ranges. Distributional characteristics were assessed descriptively using the observed range, skewness, and kurtosis. Between-group comparisons were performed using Student's *t*-test for approximately normally distributed continuous variables and the Mann–Whitney *U*-test for skewed continuous or ordinal variables. Categorical variables were compared using the chi-square test or Fisher's exact test, as appropriate. All tests were two-sided.

Radiographically confirmed gross total resection was defined as the sole primary endpoint. Operative duration, length of hospital stay, complications, neurological outcomes, and follow-up observations were considered secondary exploratory outcomes. No formal adjustment for multiple testing was applied. Consequently, *p*-values for secondary outcomes are unadjusted and should be interpreted descriptively rather than as confirmatory evidence.

Binary logistic regression was used to explore factors associated with GTR. Univariable analyses were performed for SF use, tumor location, sex, and age. An exploratory multivariable model included SF use, age, sex, and tumor location categorized as supra- vs. infratentorial. Results are reported as odds ratios with 95% confidence intervals and *p*-values. *p*-Values reported for the logistic regression models were derived from Wald tests. Fisher's exact test was used separately for the unadjusted 2 × 2 comparison of GTR proportions between the SF and non-SF groups.

Histological entity and the complete categorical World Health Organization (WHO)-grade variable were not included in the multivariable model because of marked heterogeneity between tumor entities and very small numbers in several categories, particularly WHO grades II and III. Because only 10 procedures resulted in subtotal resection, the number of outcome observations available for multivariable modeling was limited. The regression analysis was therefore considered exploratory and potentially susceptible to overfitting and residual confounding.

The surgical year was not included because SF use was closely linked to the surgical era, resulting in substantial collinearity between the treatment group and time period. Surgeon-related effects could likewise not be reliably separated from the surgical era because six surgeons contributed relatively small and temporally clustered case numbers.

Operative duration was right-skewed, particularly in the non-SF group, and was therefore compared using a two-sided Mann–Whitney *U*-test. A rank-based effect size was calculated as $r=|Z|/\sqrt{N}$. Means, standard deviations, and 95% confidence intervals of the group means were retained as complementary descriptive measures.

Because follow-up duration differed substantially between groups, recurrence and repeat-tumor-resection proportions were reported descriptively only and were not interpreted as comparative oncological outcomes.

Non-significant baseline *p*-values were not interpreted as evidence of equivalence, adequate covariate balance, or absence of confounding.

## Results

### Patient cohort and demographics

A total of 60 procedures were performed in 50 pediatric patients between 2015 and 2023, which met the inclusion criteria for this analysis.

Thirty-two cases (53.3%) underwent surgery using SF, while 28 (46.7%) were operated under white-light microscopy.

Baseline characteristics are presented in Table 1. Because of the limited sample size, non-significant between-group *p*-values were not interpreted as evidence of equivalence or adequate covariate balance.

**Table 1:** Baseline demographic and tumor characteristics.

Characteristic	Total cohort (*n* = 60)	SF group (*n* = 32)	Non-SF group (*n* = 28)	*p*-Value
Sex, *n* (%)				0.796
Male	32 (53.3)	18 (56.3)	14 (50.0)	
Female	28 (46.7)	14 (43.8)	14 (50.0)	
Age (years)				0.138
Mean	8.80	9.63	7.86	
Tumor location, *n* (%)				0.300
Infratentorial	29 (48.3)	13 (40.6)	16 (57.1)	
Supratentorial	31 (51.7)	19 (59.4)	12 (42.9)	
WHO grade, *n* (%)				0.631
I	28 (46.7)	16 (50.0)	12 (42.9)	
II	5 (8.3)	2 (6.3)	3 (10.7)	
III	4 (6.7)	1 (3.1)	3 (10.7)	
IV	23 (38.3)	13 (40.6)	10 (35.7)	

Data are presented as *n* (%) unless otherwise indicated. Between-group *p*-values are presented descriptively and should not be interpreted as demonstrating equivalence or adequate covariate balance.

SF, sodium fluorescein; WHO, World Health Organization.

Potentially clinically relevant differences were present. The SF group had a higher proportion of supratentorial tumors than the non-SF group (19/32, 59.4% vs. 12/28, 42.9%) and a somewhat higher proportion of WHO grade I tumors (16/32, 50.0% vs. 12/28, 42.9%). Conversely, infratentorial tumors were more frequent in the non-SF group (16/28, 57.1% vs. 13/32, 40.6%), and WHO grade III tumors were more frequent in the non-SF group (3/28, 10.7% vs. 1/32, 3.1%). These differences were considered potential sources of confounding for both the extent of resection and operative duration.

The most common tumor types were pilocytic astrocytomas, medulloblastomas, ependymomas, atypical teratoid/rhabdoid tumor (ATRT), and other low-grade or high-grade gliomas. Tumor localization was heterogeneous and included the posterior fossa, cerebellar hemispheres, vermis, fourth ventricle, supratentorial regions, pineal gland, tectum, and cerebellopontine angle (Table 2). The distribution of supra- and infratentorial tumors is presented in Tables 1, 2 and was considered a potential source of residual confounding. Preoperative neurological symptoms primarily consisted of signs of increased intracranial pressure, including headache and vomiting, cerebellar dysfunction such as ataxia, and cranial nerve deficits. Symptom frequencies were recorded descriptively.

**Table 2:** Tumor location.

Tumor location	SF group (*n* = 32)	Non-SF group (*n* = 28)
Supratentorial	19	12
Frontal lobe	3	2
Central region	4	1
Temporal lobe	3	4
Parietal lobe	4	3
Occipital lobe	4	3
Lateral ventricle	3	2
Infratentorial	13	16
Cerebellar	8	9
Fourth ventricle	5	6
Internal auditory canal	*0*	*1*

Six primary surgeons contributed procedures during the study period. Descriptively, the most experienced surgeons did not preferentially perform SF-guided procedures; rather, many SF-guided procedures were performed by surgeons with comparatively less experience. Because surgeon experience was not quantified using a prespecified metric and surgeon-specific case numbers were small and temporally clustered, no formal surgeon-level analysis was performed.

### Extent of resection (primary endpoint)

GTR was achieved significantly more often in the SF-guided group compared with white-light surgery (Figure 1).

**Figure 1:**
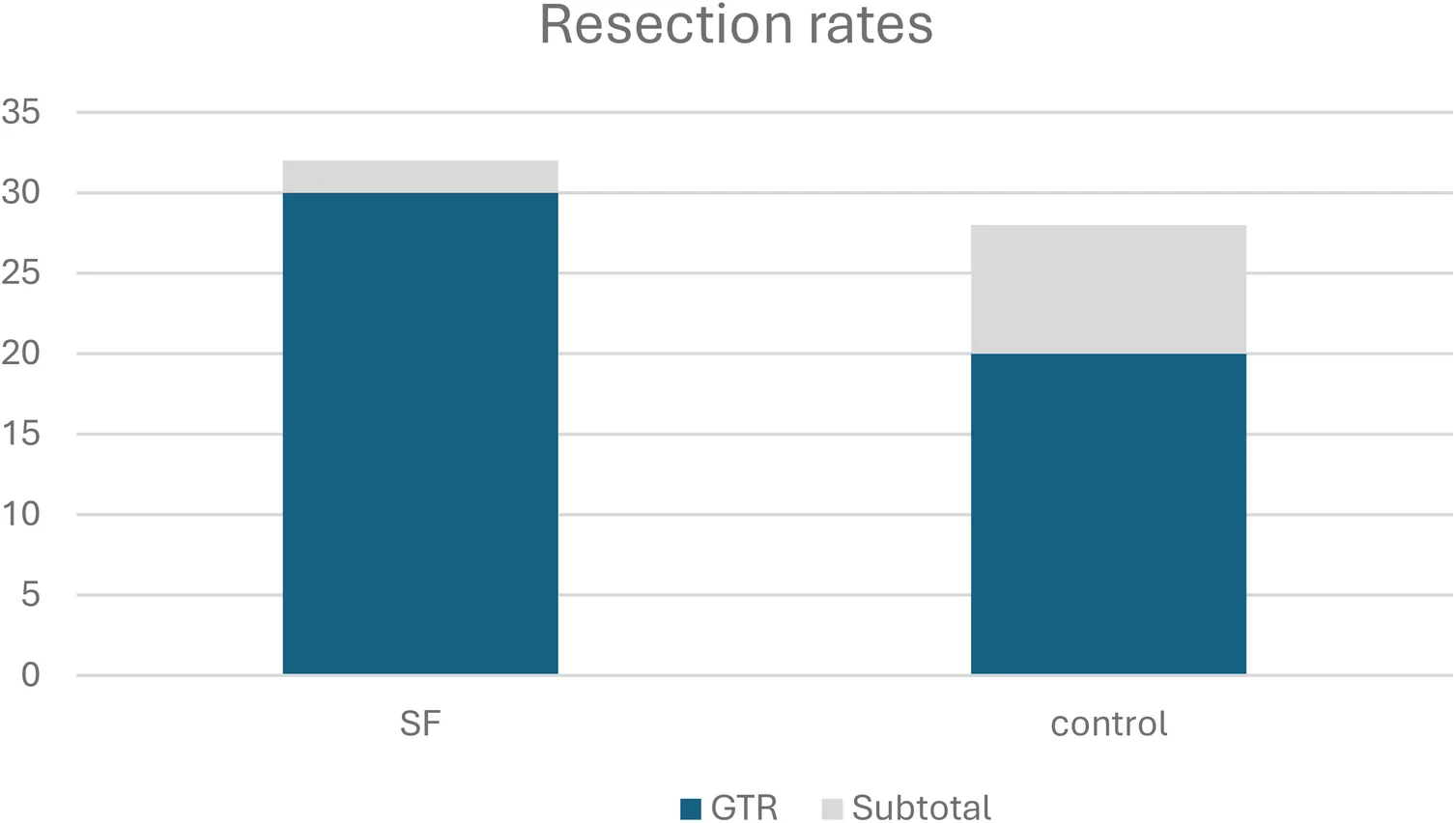
Resection rates in the sodium fluorescein group (SF) and in the control group (control). GTR, gross total resection; subtotal, subtotal resection (*p* = 0.035).

**Figure 2:**
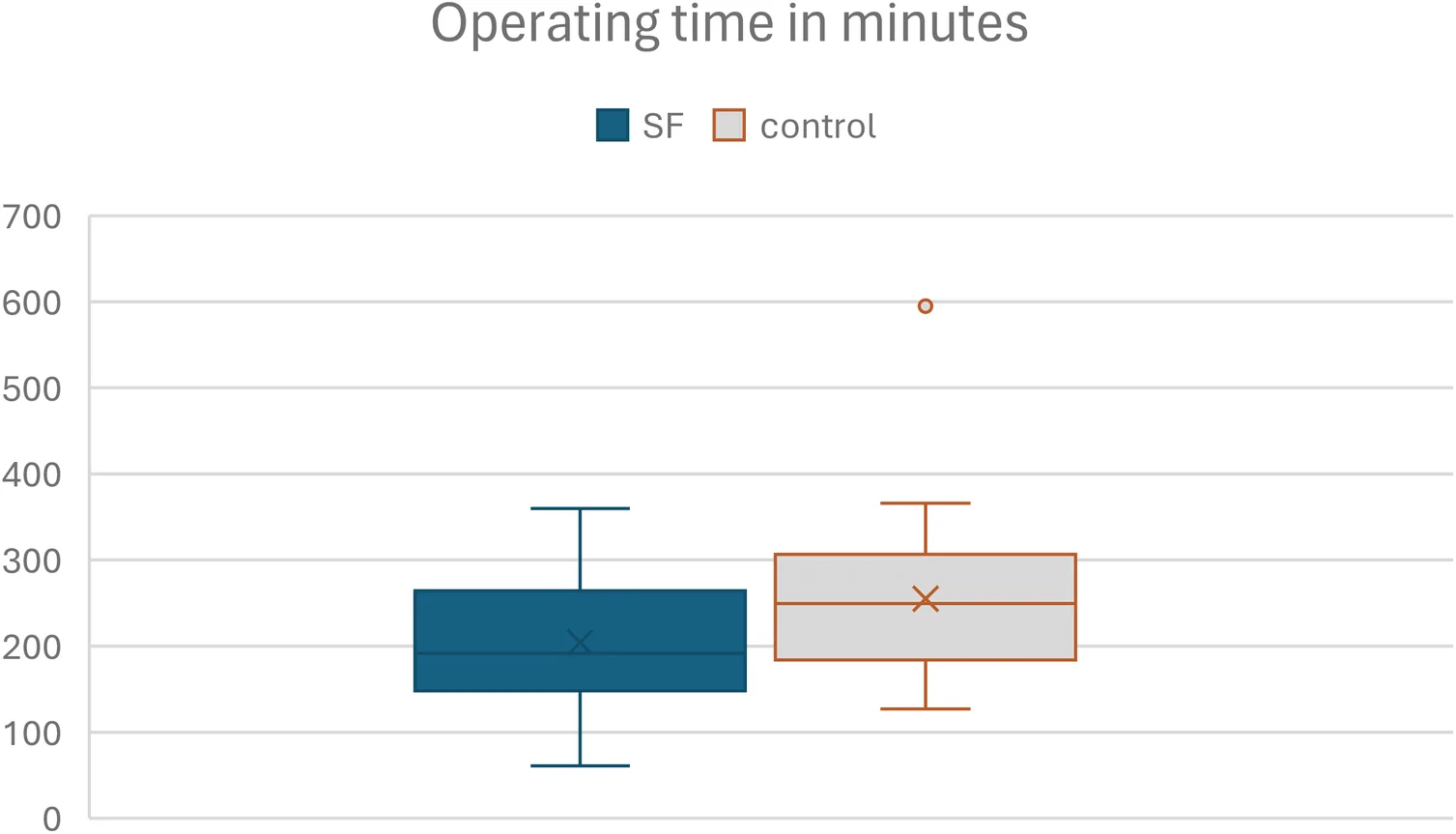
Distribution of operative duration in the sodium fluorescein and non-SF groups. Operative duration was lower in the SF group in the unadjusted exploratory Mann–Whitney *U*-test (*U* = 303, *p* = 0.032).

**Figure 3:**
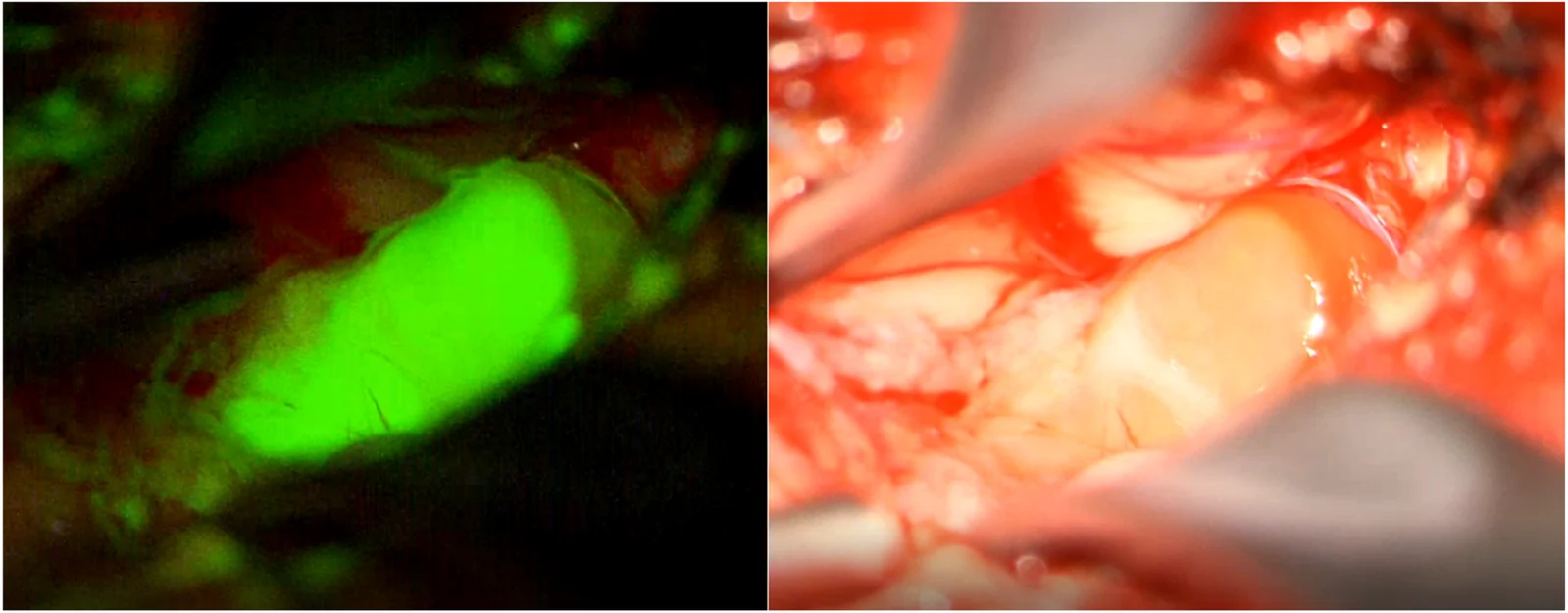
Intraoperative situs of a 7-year-old female patient with a pilocytic astrozytoma in the region of the fourth ventricle using sodium fluorescein. Left side: view under the blue light filter of the microscope; right side: view under natural light.

SF group: 93.8% GTR, 6.2% STR

White-light group: 71.4% GTR, 28.6% STR

This represents a 22.4-percentage-point increase in GTR rate associated with SF use (*p* = 0.035).

GTR was achieved in 30 of 32 SF-guided procedures (93.8%) and in 20 of 28 non-SF procedures (71.4%), corresponding to an absolute difference of 22.4 percentage points (Fisher's exact *p* = 0.035). In the univariable logistic regression analysis, SF use was associated with GTR with an odds ratio (OR) of 6.000 [95% confidence interval (CI) 1.153–31.228; Wald *p* = 0.033].

An exploratory multivariable logistic regression model was used to assess the association between SF use and GTR while adjusting for age, sex, and supra- vs. infratentorial tumor location. In this model, SF use was associated with GTR (adjusted OR 5.890, 95% CI 1.091–31.080; *p* = 0.039). Full univariable and multivariable results are presented in Table 3.

**Table 3:** Univariable and multivariable logistic regression analyses of factors associated with gross total resection.

Analysis/variable	Odds ratio (OR)	95% confidence interval	*p*-Value
Univariable analysis			
Use of SF	6.000	1.153–31.228	0.033
Tumor location: infratentorial vs. supratentorial	0.923	0.237–3.590	0.908
Sex: male vs. female	1.909	0.479–7.610	0.359
Age	0.954	0.819–1.112	0.548
Multivariable analysis			
Use of SF	5.890	1.091–31.080	0.039
Tumor location: infratentorial vs. supratentorial	1.227	0.269–5.589	0.792
Sex: male vs. female	1.788	0.417–7.673	0.434
Age	0.979	0.827–1.158	0.804

Univariable and multivariable *p*-values are Wald *p*-values derived from binary logistic regression. The two-sided Fisher's exact *p*-value for the unadjusted comparison of GTR proportions is reported separately in the Results section and is not included in this table. The multivariable model included SF use, tumor location, sex, and age. Reference categories were non-SF surgery, supratentorial location, and female sex. The analysis included 60 procedures, of which 50 resulted in GTR and 10 in STR. Given the limited number of STR outcomes and the absence of histological adjustment, the model should be interpreted as exploratory.

OR, odds ratio; CI, confidence interval; GTR, gross total resection; STR, subtotal resection; SF, sodium fluorescein.

The confidence interval was wide, reflecting the limited number of subtotal resections. Histological entity and WHO grade could not be reliably included due to the heterogeneous tumor spectrum and sparse categories. The model therefore represents a partially adjusted exploratory analysis and does not establish an independent or causal effect of SF.

### Intraoperative fluorescence characteristics (SF group)

Of the 32 SF-guided procedures, fluorescence intensity was documented in 20 procedures (62.5%). Positive fluorescence was reported in 17 of these 20 documented procedures (85.0%), corresponding to 17 of all 32 SF-guided procedures (53.1%). No fluorescence was reported in three of the 20 documented procedures (15.0%), corresponding to three of all 32 SF-guided procedures (9.4%). Fluorescence information was unavailable in the remaining 12 procedures (37.5%); missing documentation was not interpreted as absent fluorescence.

Because fluorescence documentation was incomplete and only two procedures in the SF group resulted in subtotal resection, no reliable per-case association between fluorescence intensity and radiographic extent of resection could be assessed.

Additional intraoperative adjuncts, defined as the use of neuronavigation, intraoperative ultrasound, and/or neuromonitoring, were used selectively based on the individual surgical indication. No statistically significant difference in the use of adjuncts was detected between the SF and non-SF groups (Fisher's exact test, *p* = 0.112).

### Operative parameters

#### Operative duration

Operative duration showed a right-skewed distribution, particularly in the non-SF group, with skewness 1.547 and kurtosis 4.466. Median operative duration was 191.5 min (IQR 148.25–264.25; range 61–360 min) in the SF group and 249.5 min (IQR 184.00–306.75; range 127–595 min) in the non-SF group.

Operative duration was lower in the SF group in the unadjusted exploratory Mann–Whitney *U*-test (*U* = 303, *Z* = −2.149, two-sided *p* = 0.032; rank-based effect size *r* = 0.28). The corresponding mean operative durations were 204.31 ± 70.55 min in the SF group and 254.89 ± 96.52 min in the non-SF group. Detailed descriptive results are presented in Table 4 and Figure 2.

**Table 4:** Descriptive and unadjusted between-group analysis of operative duration.

Measure	SF group (*n* = 32)	Non-SF group (*n* = 28)	Between-group result
Median (min)	191.50	249.50	
Interquartile range (min)	148.25–264.25	184.00–306.75	
Mean ± SD (min)	204.31 ± 70.55	254.89 ± 96.52	
95% CI of the mean (min)	178.88–229.75	217.47–292.32	
Range (min)	61–360	127–595	
Mann–Whitney *U*-test			*U* = 303; *Z* = −2.149; *p* = 0.032
Rank-based effect size			*r* = 0.28

Operative duration was right-skewed, particularly in the non-SF group (skewness 1.547; kurtosis 4.466) and was therefore compared using a two-sided Mann–Whitney *U*-test. The effect size *r* was calculated as |*Z*|/sqrt(*N*). This analysis was unadjusted and should be interpreted as exploratory.

CI, confidence interval; SD, standard deviation; SF, sodium fluorescein.

Because this comparison was not adjusted for tumor location, histology, surgical era, surgeon, or other measures of procedural complexity, it does not establish an independent effect of SF on operative duration.

#### Hospital stay

The median postoperative length of hospital stay for the overall cohort was 7.5 days. The mean postoperative length of stay was 9.92 ± 8.294 days (range: 4–64 days). In the SF group, the mean postoperative hospital stay was 8.69 ± 3.94 days, whereas in the non-SF control group, it was 11.32 ± 11.34 days. The difference between groups was not statistically significant [*t*(58) = −1.23; *p* = 0.223].

### Postoperative complications and safety

#### Intraoperative adverse events

No SF-related complications occurred, especially no cases of anaphylaxis or intraoperative hypotension.

Yellow discoloration of urine was observed consistently and considered clinically irrelevant.

#### Neurological outcomes

New postoperative neurological deficits occurred at similar rates in both groups (37.5% vs. 42.9%, *p* = 0.813), with most deficits being transient (e.g., cerebellar mutism, cranial nerve palsies, or ataxia). No increase in postoperative morbidity was associated with SF.

#### Postoperative complications

In the SF group, a postoperative increase in laboratory inflammatory markers (e.g., CRP or leukocyte count) was documented in six cases (18.75%), compared with five cases (17.86%, non-significant) in the non-SF control group. Due to the retrospective nature of the dataset, further infectious work-up or standardized assessment of these findings was not possible. These observations were not a primary study endpoint but were recorded descriptively and exploratorily as part of the clinical documentation.

When comparing the occurrence of at least one postoperative neurological deficit—irrespective of whether symptoms were already present preoperatively—no significant difference was observed between groups. Temporary neurological symptoms occurred in 12/32 patients (37.5%) in the SF group and in 12/28 patients (42.9%) in the non-SF control group (*p* = 0.813).

In the symptom-specific analysis (improvement of pre-existing symptoms vs. new-onset deficits), no significant between-group differences were found for most outcomes.

#### Follow-up and descriptive recurrence observations

Postoperative follow-up data were available at the first documented follow-up visit, generally approximately 3 months after surgery, and at the most recent available follow-up visit. Two of 32 SF procedures and five of 28 non-SF procedures were lost to follow-up.

The mean long-term follow-up was 29.1 ± 29.1 months in the SF group and 60.7 ± 40.3 months in the non-SF group. This difference was statistically significant (*p* = 0.046).

Repeated tumor resection during the study period was documented in seven cases in each group, corresponding to crude observed proportions of 21.9% in the SF group and 25.0% in the non-SF group. However, because follow-up was substantially shorter in the SF group, the opportunity to detect recurrence or subsequent tumor surgery was unequal. These crude proportions were therefore not subjected to comparative interpretation and cannot be used to conclude that recurrence patterns were similar between groups.

Revision surgery for postoperative complications was performed in four cases in the SF group and five cases in the non-SF group. These observations represent secondary exploratory outcomes.

## Discussion

The findings of this study indicate that low-dose SF was safe and feasible and was associated with higher GTR rates and lower operative duration in this retrospective before-and-after cohort. However, the non-randomized historical-control design, the limited sample size, and residual differences between the groups prevent causal attribution of these findings to SF alone. The unequal follow-up durations also preclude comparative conclusions regarding recurrence or long-term oncological outcomes.

### Interpretation of the radiographic extent of resection

The most prominent radiographic finding in our study was a higher GTR rate in the SF group (93.8%) than in the non-SF group (71.4%). This difference should be interpreted as an early surgical imaging endpoint rather than as evidence of improved survival or long-term oncological benefit.

The association between SF use and GTR remained statistically significant in an exploratory model adjusting for age, sex, and broad tumor location. However, the model did not include histological entity or WHO grade and was based on only 10 subtotal resections. The wide confidence interval reflects the resulting statistical uncertainty. The model should therefore not be interpreted as demonstrating an independent treatment effect.

In addition, the SF group contained a proportionally greater number of supratentorial and WHO grade I tumors, whereas the non-SF group contained a proportionally greater number of infratentorial and WHO grade III tumors. These differences may have contributed to the observed GTR difference.

Contemporary pediatric neuro-oncology increasingly relies on integrated histomolecular diagnoses, and the prognostic relevance of the extent of resection varies substantially between tumor entities and molecular subgroups. This is particularly relevant for medulloblastoma, for which Thompson et al. demonstrated that the prognostic association of extent of resection was attenuated after accounting for molecular subgroup and cautioned against aggressive removal of small residual tumors when the anticipated neurological morbidity is substantial. Accordingly, our findings should not be interpreted as showing that SF-guided surgery improves survival. Rather, they indicate an association between SF use and a higher rate of radiographically complete resection in selected gadolinium-enhancing tumors considered surgically amenable to GTR.

The higher GTR rate observed in the SF group is consistent with adult literature showing enhanced tumor boundary identification with SF-guided surgery. In adults, SF has improved resection of high-grade gliomas, metastases, hemangioblastomas, and lymphomas, often in challenging anatomical locations (13–15, 19). Our findings suggest that SF may also be useful for intraoperative visualization in selected pediatric tumors, which often exhibit strong gadolinium enhancement and are therefore well suited for SF-based visualization (see Figure 3).

### Comparison with existing pediatric literature

Previous pediatric studies have been encouraging but limited. Göker and Kırış demonstrated feasibility and intense fluorescence in the majority of their pediatric cases; however, they did not include a white-light control group. The multicenter series by de Laurentis et al. similarly showed strong fluorescence across a spectrum of pediatric tumors but also lacked matched clinical outcomes or statistical comparison. Our previously published pediatric posterior fossa dataset (19 cases) also demonstrated helpful staining in nearly 60% of cases and excellent safety, but again lacked a direct comparison group (16–18).

The present study adds a direct within-institution comparison between SF-guided and non-SF procedures. However, the historical-control design, clinically relevant baseline differences, and limited ability to adjust for tumor histology and surgical era restrict the strength of causal inference. The findings should therefore be regarded as hypothesis-generating.

### Fluorescence observations and mechanistic considerations

The mechanism of SF uptake—extravasation through a disrupted blood-brain barrier—makes it particularly well suited for pediatric neuro-oncology. Pediatric brain tumors, especially embryonal tumors (medulloblastoma, ATRT), ependymomas, and pilocytic astrocytomas, frequently exhibit strong and homogeneous contrast enhancement on preoperative MRI. This correlates closely with SF fluorescence intensity, as demonstrated in our earlier reports. In our SF group, fluorescence was reported as clearly positive in 85% of evaluable cases, including both low- and high-grade tumors (20–23).

The real-time nature of fluorescence visualization also appears advantageous during piece-meal debulking, a frequent necessity in pediatric neurosurgery. SF highlights even small remnants of enhancing tumor tissue, allowing the surgeon to identify boundaries that may be indistinguishable under white light, particularly when edema, gliosis, or postoperative bleeding obscures the interface.

### Operative duration

SF-guided procedures were associated with a shorter operative duration in the unadjusted exploratory analysis. The median operative duration was 58 min shorter in the SF group, and the rank-based effect size was *r* = 0.28. Improved visualization and more rapid identification of enhancing tumor tissue may explain this observation.

However, operative duration is influenced by numerous factors, including tumor location, histology, tumor consistency, surgical approach, case complexity, primary surgeon, senior supervision, procedural adjuncts, team composition, and surgical era. In particular, the higher proportion of infratentorial tumors in the non-SF group may have contributed to longer operative durations.

Six surgeons contributed procedures during the study period. Descriptively, many SF-guided procedures were performed by surgeons with comparatively less experience, indicating that SF cases were not preferentially assigned to the most experienced surgeons. However, this qualitative observation cannot rule out surgeon-related confounding because surgeon experience was not quantified systematically, and case selection, supervision, and tumor complexity may have differed between surgeons.

Because no adjusted analysis of operative duration was performed, the present study cannot establish that SF independently reduced operative duration.

Shorter operative duration may be clinically relevant in pediatric patients because prolonged anesthesia exposure has been associated with perioperative stress and adverse postoperative outcomes (24–26). Nevertheless, the present unadjusted analysis does not establish that SF was responsible for the observed difference.

### Safety profile

Our results reaffirm the excellent safety profile of SF in children. Across 32 SF-guided procedures, no adverse reactions such as hypotension, anaphylaxis, or hemodynamic instability occurred. This aligns with the strong safety record of SF in ophthalmology and adult neurosurgery, where millions of doses have been administered with an extremely low incidence of severe adverse reactions.

Importantly, SF did not increase postoperative complication rates, neurological deficits, revisional surgery rates, or mortality. The only postoperative death in our cohort occurred in the white-light group, related to cerebellar swelling. The absence of SF-related complications in our study is consistent with our prior 19-case series and further supports its use even in young children and infants.

### Recurrence patterns and follow-up

The follow-up duration differed substantially between the groups, with a mean follow-up of 29.1 months in the SF group and 60.7 months in the non-SF group. Consequently, patients in the SF group had a markedly shorter observation period during which recurrence or repeat surgery could be detected.

The crude observed proportions of repeat tumor resection should therefore not be interpreted as comparable recurrence rates. The present study cannot determine whether SF-guided surgery influenced recurrence, progression-free survival, or overall survival. Appropriate assessment of these outcomes would require comparable follow-up duration, time-to-event methods, standardized molecular classification, and larger entity-specific cohorts.

### Clinical integration and workflow considerations

SF integrates seamlessly into existing pediatric neurosurgical workflows. It requires no changes to anesthesia protocols, no special patient preparation, and no substantial increase in operative cost. It is compatible with neuronavigation, neuromonitoring, intraoperative ultrasound, and endoscopic techniques. The ability to toggle between white light and fluorescence within seconds enables the surgeon to incorporate fluorescence into every phase of the operation—from initial cortical entry to final cavity inspection—without disrupting the surgical flow (8).

At centers lacking intraoperative MRI or fluorescence-capable endoscopy, SF can provide a cost-effective enhancement to surgical visualization. Its affordability makes it accessible to low-resource settings globally, potentially reducing disparities in surgical outcomes.

### Limitations

Several limitations must be acknowledged. First, this is a retrospective, non-randomized study. Baseline demographic and tumor-related characteristics showed clinically relevant differences between the groups, particularly in tumor location and WHO grade. Selection bias and residual confounding therefore cannot be excluded.

Fluorescence intensity was documented in only 20 of the 32 SF-guided procedures. The reported positivity rate of 85% therefore applies only to the documented subset; positive fluorescence was documented in 17 of the 32 SF-guided procedures. Documentation was missing in 12 procedures, and the missingness may not have been random.

In addition, only two SF-guided procedures resulted in subtotal resection. Consequently, a per-case analysis of fluorescence grade vs. extent of resection would have involved very sparse outcome categories and would not have been considered reliable. The study therefore cannot determine whether fluorescence intensity was associated with GTR or whether fluorescence directly mediated the observed between-group difference.

Third, the heterogeneity of tumor types, locations, and surgical strategies—although reflective of real-world practice—limits the ability to draw tumor-specific conclusions. Finally, longer-term neurocognitive and functional outcomes were beyond the scope of this study but represent important endpoints for future research.

Although an exploratory multivariable logistic regression model was performed, the analysis was constrained by the limited number of subtotal resections. Only 10 procedures resulted in STR, whereas 50 resulted in GTR, limiting the number of covariates that could be included without a substantial risk of overfitting.

Histological entity and WHO grade could not be modeled reliably because of the heterogeneous tumor spectrum and sparse categories, particularly for WHO grades II and III. The regression model therefore provides only partial adjustment and does not eliminate residual confounding by tumor biology or baseline resectability.

Furthermore, the SF group contained a proportionally greater number of supratentorial and WHO grade I tumors, whereas the non-SF group contained a proportionally greater number of infratentorial and WHO grade III tumors. These differences may have favored both GTR and shorter operative duration in the SF group.

A multiplicity correction was not applied to the secondary outcomes. These analyses should therefore be interpreted as exploratory, and isolated secondary *p*-values should not be considered confirmatory evidence.

Follow-up was significantly shorter in the SF group. This imbalance limits the opportunity to detect recurrence and makes comparative interpretation of crude recurrence or repeat-resection proportions inappropriate.

Furthermore, molecular subgrouping and integrated WHO CNS5 diagnoses were not available in a standardized manner for all cases across the study period. Because the prognostic relevance of the extent of resection is entity- and molecular-subgroup dependent, particularly in tumors such as medulloblastoma, the higher GTR rate observed in the SF group cannot be used as a surrogate for improved survival or long-term oncological benefit.

Six primary surgeons contributed procedures during the study period, and surgeon assignment was not evenly distributed over time. Descriptively, the most experienced surgeons did not preferentially perform SF-guided procedures; many were performed by surgeons with comparatively less experience. A simple learning-curve effect favoring the SF group is therefore unlikely to fully explain the observed findings.

Nevertheless, this qualitative observation does not eliminate surgeon-related confounding. Surgeon-specific case numbers were small and temporally clustered, and surgeon assignment may have been associated with case selection, tumor complexity, operative approach, senior supervision, and other unmeasured factors. Because surgeon, surgical era, and SF use were closely interrelated, the surgeon could not be incorporated reliably into the multivariable model.

Although baseline surgical resectability was part of the inclusion criteria and tumors with anticipated unsafe complete resection due to eloquent-area involvement or other anatomical constraints were excluded, additional imaging-based variables were not systematically quantified. In particular, tumor volume, enhancement pattern, and cystic vs. solid composition may still have influenced the likelihood of achieving GTR and therefore limit causal attribution of the observed difference to sodium fluorescein alone.

Additional intraoperative adjuncts, including neuronavigation, intraoperative ultrasound, and neuromonitoring, were selected according to individual surgical indications rather than a standardized protocol. Although no statistically significant difference in the use of adjuncts was detected between the SF and non-SF groups, this finding does not establish equivalence.

Because of the limited sample size and the relatively small number of subtotal resections, a comprehensive multivariable adjustment for all potentially relevant confounders was not feasible. The observed associations between sodium fluorescein use, GTR rate, and operative duration should therefore be interpreted cautiously and cannot establish a causal treatment effect.

Most importantly, the study represents a before-and-after comparison with historical controls rather than a contemporaneous controlled design. SF use was therefore closely linked to the surgical era, and temporal confounding cannot be excluded. Changes in surgical workflow, team composition, primary operating surgeon, degree of senior supervision, perioperative management, institutional experience, and available adjunctive technologies over the 8-year study period may have influenced both GTR rates and operative duration.

Because the treatment group and surgical era were highly collinear, reliable adjustment for year of surgery was not feasible in this limited cohort. The observed differences should therefore be interpreted as associations rather than evidence of a causal SF effect.

### Future directions

Prospective multicenter trials with larger sample sizes, standardized fluorescence scoring, molecular tumor stratification, and long-term follow-up are needed to confirm these findings. The integration of SF with emerging technologies—such as fluorescence-enabled neuroendoscopy, augmented-reality navigation, and quantitative fluorescence imaging—represents an exciting avenue for further refining intraoperative tumor visualization.

## Conclusion

In this retrospective, before-and-after, single-center cohort, low-dose sodium fluorescein was safe and feasible and was associated with higher rates of radiographically confirmed gross total resection and shorter operative duration in selected gadolinium-enhancing pediatric brain tumors.

However, the historical-control design, limited number of non-GTR outcomes, clinically relevant baseline differences in tumor location and WHO grade, heterogeneous tumor spectrum, unequal follow-up, and incomplete adjustment for histology, surgical era, and surgeon-related factors prevent causal or long-term oncological conclusions.

The results should therefore be considered exploratory and hypothesis-generating and require prospective validation in larger, preferably multicenter cohorts with standardized radiological, histomolecular, operative, and follow-up variables.

## Data Availability

The raw data supporting the conclusions of this article will be made available by the authors, without undue reservation.
